# Relationship between distal radius fracture malunion and arm-related disability: A prospective population-based cohort study with 1-year follow-up

**DOI:** 10.1186/1471-2474-12-9

**Published:** 2011-01-13

**Authors:** Elisabeth Brogren, Manfred Hofer, Michael Petranek, Philippe Wagner, Lars B Dahlin, Isam Atroshi

**Affiliations:** 1 Department of Clinical Sciences, Malmö-Hand Surgery, Lund University, Lund, and Department of Hand Surgery, Malmö University Hospital, Malmö, Sweden; 2 Department of Physical and Occupational Therapy, Kristianstad Hospital, Kristianstad, Sweden; 3 Department of Radiology, Hässleholm Hospital, Hässleholm, Sweden; 4 Swedish National Competence Center for Musculoskeletal Disorders, Department of Orthopedics, Lund University Hospital, Lund, Sweden; 5 Department of Clinical Sciences, Lund, Lund University, Lund, and Department of Orthopedics Hässleholm-Kristianstad, Hässleholm Hospital, Hässleholm, Sweden

## Abstract

**Background:**

Distal radius fracture is a common injury and may result in substantial dysfunction and pain. The purpose was to investigate the relationship between distal radius fracture malunion and arm-related disability.

**Methods:**

The prospective population-based cohort study included 143 consecutive patients above 18 years with an acute distal radius fracture treated with closed reduction and either cast (55 patients) or external and/or percutaneous pin fixation (88 patients). The patients were evaluated with the disabilities of the arm, shoulder and hand (DASH) questionnaire at baseline (concerning disabilities before fracture) and one year after fracture. The 1-year follow-up included the SF-12 health status questionnaire and clinical and radiographic examinations. Patients were classified into three hypothesized severity categories based on fracture malunion; no malunion, malunion involving either dorsal tilt (>10 degrees) or ulnar variance (≥1 mm), and combined malunion involving both dorsal tilt and ulnar variance. Multivariate regression analyses were performed to determine the relationship between the 1-year DASH score and malunion and the relative risk (RR) of obtaining DASH score ≥15 and the number needed to harm (NNH) were calculated.

**Results:**

The mean DASH score at one year after fracture was significantly higher by a minimum of 10 points with each malunion severity category. The RR for persistent disability was 2.5 if the fracture healed with malunion involving either dorsal tilt or ulnar variance and 3.7 if the fracture healed with combined malunion. The NNH was 2.5 (95% CI 1.8-5.4). Malunion had a statistically significant relationship with worse SF-12 score (physical health) and grip strength.

**Conclusion:**

Malunion after distal radius fracture was associated with higher arm-related disability regardless of age.

## Background

Patients with a distal radius fracture generally recover within six months after fracture, although a minority of patients experience prolonged functional impairment and pain [1]. Current management of displaced distal radius fractures includes various surgical and non-surgical methods that are, however, based on limited evidence [2]. One basic question concerns the degree to which functional outcome is related to the radiological appearance after fracture union. The likelihood of anatomical restoration may improve with the use of treatment methods that, compared with closed reduction and splinting, may require more advanced surgical training and equipment probably resulting in increased costs. However, this may not be justified if functional outcome is not substantially improved. A number of studies have suggested that a strong relationship exists between anatomical restoration and function after distal radius fracture, whereas others have reported acceptable functional outcome regardless of radiographic deformity, especially among the elderly [3-7]. Most previous studies were not population-based and few used validated patient-reported outcome measures. Thus, the yet unresolved question of the relationship between functional outcome and malunion after these fractures needs further evaluation.

We conducted a prospective population-based cohort study of patients with distal radius fracture to investigate the relationship between fracture malunion and arm-related disability at 1 year after fracture.

## Methods

### Study design and population

From January 2001 through March 2002 we prospectively enrolled consecutive patients with distal radius fracture at one emergency department in northeastern Scania health district in southern Sweden. The orthopedic department is the only facility in that health district where closed or open reduction of distal radius fractures is performed and all patients were treated at this facility. The inclusion criteria for the present study were acute fracture of the distal radius treated with closed reduction and cast or with closed reduction and external fixation or percutanoues pin fixation and patient age above 18 years. The exclusion criteria were residence outside the region at the time of fracture according to the national population register, severe medical illness or cognitive disorder precluding participation in the follow-up examination, unwillingness to participate, treatment with open reduction and internal fixation (ORIF), and death within one year from fracture date. The study was approved by the Regional Ethical Review Board and informed consent was obtained from each patient.

Data concerning type of trauma were obtained. Fall at the same level from an upright position was classified as moderate trauma and all other types of trauma (falling from heights, traffic accident or trauma during exercise) were classified as severe trauma.

**Figure 1:**
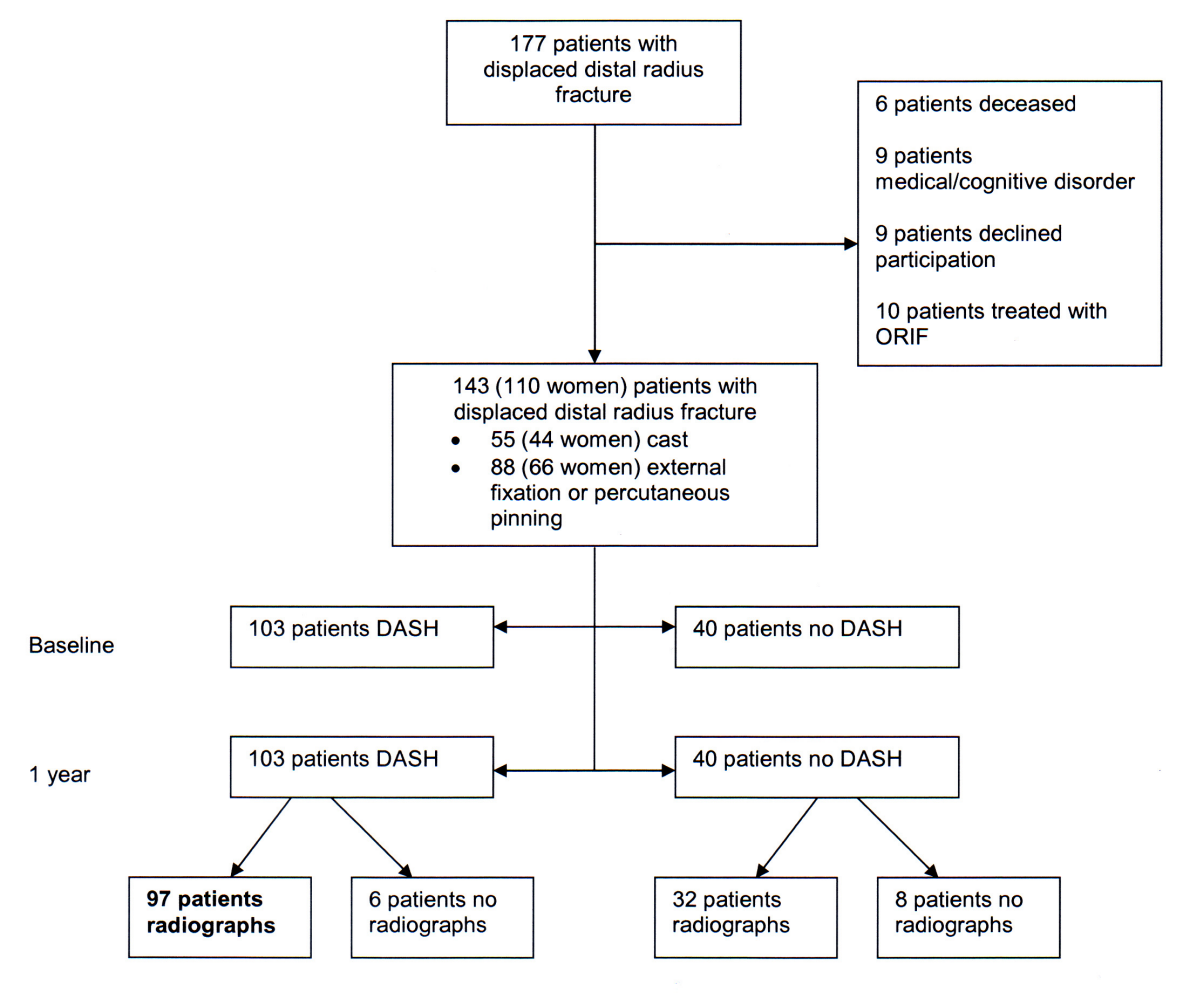
**Flowchart of patients with displaced distal radius fracture and their follow-up with DASH score and radiographic examination**.

Nine patients were excluded because of severe medical illness or cognitive disorder, nine patients declined to participate, 10 patients were excluded because of treatment with ORIF and six patients died within one year from fracture date. A total of 143 patients (110 women and 33 men), mean age 65 years (SD 15, range 19-95 years) participated in the study (Figure 1). The treatment method was closed reduction and cast in 55 patients (44 women), mean age 66 years (SD 16, range 19-95) and closed reduction and fixation in 88 patients (66 women), mean age 64 (SD 14, range 25-89). In the closed reduction and fixation group, 72 were treated with external fixation, six with external fixation and percutaneous pinning and 10 percutaneous pinning only. Five patients were initially treated with closed reduction and cast but subsequently underwent external fixation because of fracture re-displacement shown at the 1-week radiographic examination. None of these fractures needed additional pin fixation. The injury trauma was moderate in 95 (66.4%) patients, severe in 46 (32.2%) patients, and unknown in two patients.

### Treatment methods

The treatment method was decided by the attending orthopedic surgeon based on the clinical evaluation of the patient and the radiographic appearance of the fracture. At the time of the study the department used mainly two methods for treating displaced distal radius fractures; closed reduction and splinting, preferred for less severely displaced and comminuted fractures, and closed reduction and external or percutaneous pin fixation, preferred for fractures with severe displacement, comminution and instability. Fractures treated with closed reduction and cast were reduced at the emergency department using local anesthesia (xylocaine hematoma block) and manipulation under fluoroscopy. Patients requiring surgical fixation were treated at the operating room under regional or general anesthesia. Radiographic examination was done one week after initial treatment. Fractures that were judged to have redisplaced at one week were considered for closed re-reduction and external fixation. The decision to perform secondary external fixation was made by the treating orthopedic surgeon after discussion with the patient.

### Assessment of disability

The patients completed the disabilities of the arm, shoulder and hand (DASH) questionnaire, sent by mail, at one week (questionnaire inquired about disabilities before the fracture) and at one year after the fracture. The DASH questionnaire (30 items measuring disability and symptoms related to the upper extremity) is scored from 0 to 100 with higher score indicating higher disability [8]. The validated Swedish version of the DASH was used in this study and questionnaires with more than three unanswered items were excluded [9]. The DASH has previously been used as an outcome measure after distal radius fracture [10]. The minimum clinically important difference in DASH score has been estimated to 10 points in two previous studies involving patients with various upper extremity conditions [11,12]. Population norms for the US general population have been published, but no Swedish population norms are available [13].

The patients also completed the SF-12 health status questionnaire during the 1-year clinical examination at the hospital. The SF-12 questionnaire has 12 items and produces a physical component score (PCS) and a mental component score (MCS), each normalized to mean of 50 and standard deviation of 10 compared to the general US population, with higher score indicating better quality of life [14].

### Clinical examination

At one year after the fracture the patients were examined by a physiotherapist (MH) who measured grip strength with the Jamar^® ^Hydraulic Hand Dynamometer, Sammons Preston, Inc. Bolingbrook, IL, USA and range of motion (flexion, extension, supination and pronation) with a goniometer. The measurements were done on both arms.

### Radiographic examination

Standard posteroanterior and lateral radiographs were obtained before treatment (to verify the diagnosis) and at one week after treatment. At one year after fracture radiographic examination of both wrists was done. At the conclusion of the study an experienced radiologist (MP) with no knowledge of the patient's DASH responses classified the fractures according to the AO system and precisely measured dorsal tilt (degrees), ulnar variance (mm) with 1-mm intervals, radial inclination (degrees) and articular step-off (mm). Dorsal tilt was measured on the lateral view as the angle between a line connecting the dorsal and volar lips of the distal radius and a line perpendicular to the central axis of the radius, as described by Goldfarb et al [15]. Ulnar variance was measured on the posteroanterior view with a horizontal line drawn from the ulnar side of the mid-articular surface of the distal radius toward the ulna. Variance was determined as the distance between this line and the carpal surface of the ulna; this technique is a modification of the method described by Steyers and Blair [16]. Radial inclination was determined on the posteroanterior view as the angle between a line drawn from the distal tip of the radial styloid to the distal sigmoid notch and a line perpendicular to the long axis of the radius [15]. A resident in hand surgery (EB) independently double-checked the radiologist's measurements of the 1-year radiographs of both the injured and the uninjured wrist in a random sample of 54 patients (38%). The reliability was assessed with the intraclass correlation coefficient (ICC) and its 95% confidence interval (CI). For the radiographs of the injured wrist the ICC (95% CI) for volar tilt was 0.98 (0.96-0.99) and for ulnar variance 0.94 (0.89-0.96) and for the radiographs of the uninjured wrist it was 0.99 (0.98-0.99) and 0.88 (0.78-0.93), respectively, indicating high reliability.

### Nonrespondents

The DASH was missing both at baseline and at one year in 40 patients (28%) (Figure 1). Compared with baseline-DASH respondents (mean age 63 years, 79% women), the baseline-DASH nonrespondents (mean age 65 years, 73% women) were more likely to have had AO type C fracture (30% vs 25%) and to have been treated with surgery (75% vs 58%).

Twenty-five patients (17%) did not attend clinical examination. The baseline radiographs of nine patients and the 1-year radiographs of 14 patients were missing. The 1-year nonrespondents (46 patients with no DASH and/or radiographs at one year) did not differ significantly from the 1-year respondents (97 patients) with regard to age, sex, treatment method or fracture type (Table 1). Of the 46 1-year nonrespondents, 32 patients had attended radiographic examination at one year, but had not completed the DASH and six patients completed the DASH and did not attend radiographic examination (Figure 1). The 1-year SF-12 was missing in five respondents.

**Table 1:** Non-respondent analysis (patients with missing 1-year DASH and/or 1-year radiographs)

	Respondents N = 97	Nonrespondents N = 46	p-value
Age, mean (SD) years	64 (14)	67 (15)	0.145
Women	75 (77)	35 (76)	0.870
Cast	41 (42)	14 (30)	0.174
AO type C fracture	27 (28)	14 (30)	0.458

Results shown as n (%) unless specified otherwise

### Statistical analysis

For continuous data, means, standard deviations (SD) and medians were calculated. In all analyses 95% CI were calculated when appropriate. The primary outcome was the DASH score at one year after fracture. To assess the relationship between the radiographic variables measured and disability, we performed multiple linear regression analyses with the DASH score at one year as a dependent continuous variable and each of the variables dorsal tilt, ulnar variance and radial inclination at one year as independent continuous variable, adjusting for age, sex, fracture AO type, treatment method and the corresponding radiographic variable in the uninjured wrist. Of the radiographic variables, dorsal tilt and ulnar variance were found to have statistically significant effect on the DASH score (Table 2), whereas radial inclination had no statistically significant effect (average change per unit -0.212; 95% CI -0.94-0.52, p = 0.564). The two significant radiographic variables were further analyzed as dichotomized categorical variables (dorsal tilt ≤10° or >10° and ulnar variance ≤0 mm or ≥1 mm). When judging malunion in clinical practice, cut-off values for radiographic variables are commonly employed and recommendations based on several biomechanical and clinical studies have suggested that a dorsal tilt exceeding 10° should not be accepted [4,17-19]. Recommendations regarding the degree of ulnar variance that may be considered acceptable are more diverse and range from a positive variance of 1 mm up to 6 mm [20,21]. We chose to consider ulnar variance of 1 mm or more as malunion because no evidence suggests that only greater incongruity of the distal radioulnar joint is important with regard to disability. These cut-off values were used to classify patients into three hypothesized malunion severity categories; no malunion with both a dorsal tilt ≤ 10° and ulnar variance ≤ 0 mm, malunion involving either a dorsal tilt >10 degrees or an ulnar variance ≥1 mm, and combined malunion involving both these levels of dorsal tilt and ulnar variance.

**Table 2:** Multiple linear regression analysis of mean DASH score at one year

	Average change per unit† (95% Confidence intervals)	p-value
**Dorsal tilt**		

Age	0.198 (-0.07-0.47)	0.151
Sex	-9.81(-18.9-(-0.50))	0.035
Treatment	4.91 (-2.87-12.7)	0.213
Fracture AO type	2.90 (-1.24-7.03)	0.167
Dorsal tilt 1 year	-0.42 (-0.80-(-0.05))	0.026
Dorsal tilt uninjured hand	-0.07 (-0.49-0.35)	0.744

**Ulnar variance**		

Age	0.11 (-0.20-0.41)	0.479
Sex	-8.86 (-18.0-0.50)	0.060
Treatment	3.94 (-4.01-11.3)	0.312
Fracture AO type	2.33 (-1.66-6.6)	0.268
Ulnar variance 1 year	1.46 (0.16-2.89)	0.034
Ulnar variance uninjured hand	-1.29 (-3.42-1.05)	0.257

† adjusting for age, sex, fracture AO type, treatment method and dorsal tilt or ulnar variance of uninjured hand

For our primary outcome variable (1-year DASH score) we used analysis of covariance (ANCOVA) to calculate the mean differences in DASH scores between patients with dorsal tilt ≤ 10° versus >10° and patients with ulnar variance ≤ 0 mm versus ≥1 mm as well as between the three malunion severity categories adjusting for age, sex, and treatment method. In addition, we performed a fixed-time Cox regression analysis (robust variance) with the 1-year DASH score as the dichotomized (≥15 and <15) dependent variable and age, sex, fracture AO type, treatment method, and malunion severity category as covariates[22]. We calculated the relative risk (RR) of having the higher DASH score for each malunion severity category. We also calculated the number needed to harm (NNH) associated with malunion using a DASH score of 15 or higher as a cut-off for higher disability [23]. In choosing a DASH score of 15 as a cut-off for higher disability we took into consideration the characteristics of our fracture population and the sex and age-specific DASH population norms (mean score 10 and SD 15 for the adult population, higher scores for women than for men, successively increasing mean score and standard deviation with higher age groups, and higher score with comorbidities) [13].

For the analysis of the secondary outcomes, we used ANCOVA to determine the association between the SF-12 scores at one year and the malunion severity category adjusting for age, sex, and treatment method (analysis comprised 120 patients with 1-year radiographic and SF-12 data). Similarly, the relationships between grip strength and forearm supination and the malunion severity category were analyzed with ANCOVA adjusting for age, sex, treatment method, dominance of the injured hand, and contralateral grip strength or supination (analysis comprised 117 patients with 1-year radiographic and physical examination data). Additional analyses with adjustment also for radiographic parameters in the uninjured wrist did not change the results. All statistical tests were 2-sided and a p-value below 0.05 was considered to indicate statistical significance.

## Results

### Disability and malunion

The mean DASH score showed worsening from baseline (before injury) to one year and was similar in both treatment groups (Table 3). The external or percutaneous pin fixation group had more severe fracture displacement (dorsal tilt and ulnar variance) than the cast group at baseline, but achieved better anatomical position at one year.

**Table 3:** Radiographic measures and DASH score at baseline and one year after distal radius fracture

	All patients (n = 143)		Closed reduction and cast (n = 55)		Closed reduction and fixation* (n = 88)	
	mean (SD)	median	mean (SD)	median (range)	mean (SD)	median (range)
Dorsal tilt (degr)						
Baseline	19 (17)	19	15 (11)	15 (-4-46)	22 (19)	22 (-50-56)
1 year	4 (11)	4	8 (5)	7 (-11-29)	0 (10)	2 (-25-32)
Contralateral wrist^†^	-7.9 (8)	-10	-7.9 (6)	-8 (-16-17)	-8 (10)	-10 (-25-40)
Ulnar variance (mm)						
Baseline	2.4 (4.3)	3.0	1.6 (4.7)	3.0 (-28-12)	3.0 (3.6)	3.0 (-12-16)
1 year	2.6 (3.4)	2.0	3.5 (3.4)	3.0 (-3.0-10)	2.0 (3.4)	2.0 (-5.0-11)
Contralateral wrist^†^	-0.04 (1.7)	0	0.07 (1.7)	0 (-4.0-4.0)	-0.11 (1.8)	0 (-5.0-5.0)
Radial inclination (degr)						
Baseline	15 (8)	16	17 (7)	17 (-17-33)	14 (7)	14 (-15-34)
1 year	18 (6)	18	16 (5)	15 (5-27)	19 (6)	19 (2-36)
Contralateral wrist^†^	22 (4)	22	22 (3)	22 (12-28)	22 (4)	23 (10-31)
DASH score						
Baseline	5 (9)	0	6 (11)	0 (0-37)	4 (8)	0 (0-34)
1 year	19 (18)	14	19 (18)	15 (0-57)	19 (18)	13 (0-76)

*External fixation or percutaneous pinning

^†^Available for 115 patients

The mean DASH score (adjusted for age, sex and treatment method) was significantly worse in patients with dorsal tilt >10° than those with dorsal tilt ≤10° (adjusted mean difference 10.5, 95% CI 2.1-19.0; p = 0.015) and in patients with ulnar variance of ≥ 1 mm than in patients with ulnar variance of ≤ 0 mm (adjusted mean difference 8.7, 95% CI 0.7-16.7; p = 0.034) (Table 4). The three malunion severity categories did not differ significantly in mean baseline DASH score as analyzed with ANCOVA adjusting for age, sex, AO fracture type and treatment method (p = 0.24). The mean 1-year DASH score was significantly lower in patients with no malunion and in those with malunion involving only dorsal tilt or ulnar variance than that in patients with combined malunion; the adjusted mean differences were 17.3 (95% CI 6.5-28; p = 0.002) and 11.1 (95% CI 1.9-20.3; p = 0.019), respectively (Table 4). There were three patients with volar tilt ≥20°; however, exclusion of these patients did not change the results. Twenty-seven patients healed with no malunion. Six of them had a registered complication (median DASH 5.4) and 19 patients had no complication (median DASH 5.0).

**Table 4:** Relationship between malunion and the DASH score one year after distal radius fracture

Variables	N (%)	DASH score mean (SD)	Adjusted mean Difference (95% CI)*	p-value
Dorsal tilt				
> 10°	24 (25)	25.8 (18)	10.5 (2.1-19.0)	0.015
≤ 10°	73 (75)	16.7 (17)		
Ulnar variance				
≥ 1 mm	66 (68)	22.4 (18)	8.7 (0.7-16.7)	0.034
≤ 0 mm	31 (32)	11.6 (16)		
Malunion^†^:				
No malunion	27 (28)	11.5 (17)	17.3 (6.5-28.0)	0.002
Malunion I	50 (51)	19.1 (17)	11.1 (1.9-20.3)	0.019
Malunion II	20 (21)	28.5 (19)	Referent	

*Analysis of covariance adjusting for age, sex and treatment method (adjustment for contralateral dorsal tilt and ulnar variance among the 89 patients with contralateral wrist radiographs gave similar results with slightly larger mean differences and lower p values)

^†^No malunion, dorsal tilt ≤ 10° and ulnar variance ≤ 0 mm; Malunion I, dorsal tilt > 10° or ulnar variance ≥ 1 mm; Malunion II (combined malunion), dorsal tilt > 10° and ulnar variance ≥ 1 mm

The fixed-time Cox regression analysis adjusting for age, sex, AO fracture type and treatment method showed that patients with malunion involving either dorsal tilt or ulnar variance, or both, were significantly more likely to have higher disability (DASH score ≥15) compared to patients with no malunion (Table 5); for malunion involving either dorsal tilt >10° or ulnar variance ≥1 mm the RR was 2.5 (95% CI 1.08-5.8), and for malunion involving both dorsal tilt and ulnar variance the RR was 3.7 (95% CI 1.5-9.1). The NNH was 2.5 (95% CI 1.8-5.4).

**Table 5:** Cox regression analysis of the relationship between malunion and disability (DASH score of 15 or higher) one year after distal radius fracture

Variables	Relative risk (95% CI)	p-value
Malunion*:		
Malunion I	2.5 (1.08 - 5.8)	0.033
Malunion II	3.7 (1.5 - 9.1)	0.004
Age (year)	1.02 (1.00 - 1.03)	0.082
Sex (male)	0.43 (0.21 - 0.88)	0.020
Fracture AO type (A)	1.13 (0.75 - 1.7)	0.563
Treatment (cast)	1.36 (0.89 - 2.1)	0.151

*No malunion, dorsal tilt ≤ 10° and ulnar variance ≤ 0 mm; Malunion I, dorsal tilt > 10° or ulnar variance ≥ 1 mm; Malunion II (combined malunion), dorsal tilt > 10° and ulnar variance ≥ 1 mm

The mean SF-12 PCS score for the patients with combined malunion (n = 21) was 39.3 (SD 9), for those with malunion involving either dorsal tilt or ulnar variance (n = 65) was 45.2 (SD 10) and for those with no malunion (n = 34) was 47.9 (SD 10); the mean differences (adjusted for age, sex and treatment method) were 6.0 (95% CI 0.98-11.0; p = 0.020) and 6.8 (95% CI 1.0-12.6; p = 0.021), respectively. There were no differences in mean SF-12 MCS scores according to severity category of malunion (data not shown).

About 28% of the fractures were intraarticular according to the AO fracture classification (two type B and 38 type C fractures). Among patients that were treated with closed reduction and cast, 18% of the fractures were AO fracture type C, in comparison with 32% among the patients that were treated with external fixation and/or percutaneous pinning. Intra-articular step-off at one year was present in nine of the 97 1-year respondents (5 patients had a 0.5-mm step-off and four patients had a 1-mm step-off); their mean 1-year DASH score was 22 (SD 17, range 0-55).

Of the 32 1-year nonrespondents who had attended radiographic examination, but had not completed the DASH questionnaire, eight (25%) had no malunion, 19 (59%) had malunion involving either dorsal tilt or ulnar variance, and five (16%) had a combined malunion (p = 0.66 compared with the respondents). The mean DASH score for the six patients who did not attend the 1-year radiographic examination was 13.3 (SD 15).

### Physical measures

The mean grip strength for patients with combined malunion (n = 21) was 19.2 (SD 7) kg, for those with malunion involving either dorsal tilt or ulnar variance (n = 63) was 24.7 (SD 11) kg, and for those with no malunion (n = 33) was 33.2 (SD 16) kg; the adjusted mean differences were 3.3 (95% CI 0.08-6.6; p = 0.045) and 5.6 (95% CI 1.7-9.5; p = 0.005), respectively. In a similar analysis no statistically significant differences in supination were found.

### Complications

Complications were recorded in 47 of the 143 patients. Superficial pin tract infection requiring antibiotics occurred in 32 of the 88 patients in the external fixation and percutaneous pinning group. Osteomyelitis was diagnosed in one patient with external fixation. Carpal tunnel syndrome occurred in 10 patients (six patients in the cast group), of whom seven were treated with carpal tunnel release (two in the cast group) and three with wrist splint. Rupture of the extensor pollicis longus tendon occurred in one patient in each treatment group (one was treated with tendon transfer and one declined surgery). One patient in the external fixation and percutaneous pinning group developed chronic regional pain syndrome. One patient in the cast group developed a thumb adduction contracture that recovered with physiotherapy.

## Discussion

Our data suggest that after a distal radius fracture in adults there is a statistically significant relationship between radiological appearance at one year and patient-reported outcomes measured with the DASH questionnaire. We found that fracture malunion, defined as dorsal tilt >10° and/or ulnar variance ≥1 mm, was associated with higher disability. The mean DASH score increased by a minimum of 10 points with increased malunion severity category; a magnitude of change shown to be clinically important [11,12]. The relative risk of persistent disability (defined as DASH score ≥15) one year after a distal radius fracture increased substantially with both severity categories of malunion. The number needed to harm (NNH) in our study indicated that of every five patients with any of the two severity categories of malunion, higher disability (DASH score ≥15) would be recorded in two patients who would otherwise have had lower disability. Additionally, analysis of other outcomes also showed statistically significant worse SF-12 PCS score and weaker grip strength with every malunion severity category. Altogether, this indicates that the risk of prolonged disability after distal radius fracture would be reduced with better anatomical reconstruction.

In some previous studies no association was found between radiographic results and various outcome measures [24,25]. Souer et al. studied 84 patients recovering from distal radius fracture (majority treated with ORIF) and suggested that pain dominates the patient's perception of function measured with the DASH and that radiographic measures did not correlate with outcome. However, patients were evaluated at various stages of recovery (six to 60 months, about 30% at less than 12 months) and all fractures were treated operatively; thus, a substantial malunion was probably less common [24]. In a study of distal radius fracture treated with ORIF (volar plate), Chung et al. found that only age and income were significantly associated with 1-year outcomes, measured with the Michigan Hand Questionnaire. However, as the authors stated, most of their patients had near anatomic reduction, limiting the variability required for finding predictive factors [25]. A large proportion of patients in our study healed with malunion of varying severity which facilitates the assessment of the relationship with disability.

Although our study was not primarily designed to compare treatment methods it showed that the group treated with closed reduction and external fixation or percutaneous pinning had more severe fracture displacement at the initial postfracture radiographs, but achieved better radiological position at one year than the cast group. Accordingly, surgical fixation was more effective than cast in maintaining fracture position. However, fixation was more effective in restoring dorsal tilt than ulnar variance, which may partly explain that the unadjusted mean 1-year DASH score was similar in both treatment groups. The DASH score at baseline showed minimal disability, but had worsened in both groups at one year. There was also a difference in anesthetic block between the two treatment groups, because unlike regional anesthesia hematoma block does not provide muscle relaxation. Comparison of the two groups should be interpreted cautiously because the surgeons selected the treatment based on severity of the fracture. Therefore, the type of treatment was adjusted for in all analyses. The purpose of the study was not to compare the effectiveness of the treatments, but to assess the relationship between malunion and disability.

It is commonly believed that fracture of the distal radius causes more disability in young adults, assumed to have higher physical demands on the wrists, than in the elderly believed to better tolerate deformity. Fracture malunion has been shown to associate with higher disability among young and middle-aged adults in several studies [3,26,27]. In contrast, studies of distal radius fracture in elderly patients with comorbidities and low functional demands have shown poor correlation between radiographic and functional outcome [5-7]. Our results were adjusted for age and still showed changes in DASH score with increased malunion severity category. Assuming a similar malunion category and other characteristics, age was associated with a higher relative risk (though not statistically significant) of worse 1-year DASH score.

It is generally accepted that treatment of a distal radius fracture should aim at achieving the best possible anatomical reduction in the young active patient and at minimizing the interventions in the low-demand elderly patient with multiple comorbidities [5]. However, clinical decision-making when treating an older, but yet active, patient is more challenging. Grewal and Macdermid followed 216 patients with extra-articular fractures and found that patients at all ages had higher risk of poor functional outcome if their fracture healed with malalignment compared to those with acceptable malalignment [26]. A recently published literature review suggested that elderly patients with higher functional demands would benefit from fracture stabilization with volar locking plates [28]. Our finding that disability correlates with the severity of malunion independently of age is supportive of this proposal and may imply that functional status rather than age needs to be taken into consideration and that surgical intervention ought to be considered more frequently in all age groups.

Our study has limitations. The response rate to the DASH questionnaire was about 70%. However, the patients who failed to respond did not differ substantially from the patients who responded, which suggests that the potential influence of the loss to follow-up is not likely to be substantial. The reasons why patients did not attend follow-up are unknown. In the 1-year analyses, we used a cut-off for the DASH score of 15 to indicate higher disability. Because only a small proportion of the patients achieved 1-year scores that were as low as the baseline scores the use of a higher cut-off was justified. The US population norms for the DASH have been estimated as a mean score of 10 and standard deviation of 15 for the whole population above 18 years of age; with the mean score being higher in women than in men and gradually increasing in successive age groups [13]. We therefore believe that a score of 15 was a reasonable cut-off, but it needs to be evaluated in future studies.

Another limitation is that the DASH score may reflect disability related to upper extremity disorders other than the wrist. However, this should not differ with the severity category of malunion. We included both extra- and intra-articular fractures which could be considered a disadvantage. However, patients with an intra-articular step-off were few and their mean DASH score was only slightly higher than that for the whole patient population. Thus, it would not have a substantial effect on the results. Other factors, such as articular gap that may potentially affect disability, were not recorded, but similar to articular step-off it does not seem to be a factor that would have substantially affected the results. We did not screen for previous injuries to the contralateral wrist, the radiographic variables of which were adjusted for in our multivariate analyses. However, the mean and median values for the radiographic variables of the uninjured wrist were within normal range, suggesting that the prevalence of prior contralateral wrist fracture ought to be low and would not substantially affect the results.

We did not use a specific wrist score in this study. The DASH score has been shown to be a reliable and valid evaluation tool of outcome after distal radius fracture [10]. Correlation between the DASH score and Mayo wrist score has been reported to be strong among patients with wrist arthrodesis, and moderate among patients with surgically treated distal radius fractures [24,29]. Because the purpose of our study was to assess the relationship between malunion and disability we chose to use the DASH score as a widely used patient-reported outcomes measure of upper-extremity related disability. Although a wrist score also would be of interest we believe it represents a different type of outcome. In addition, we did not take into account the effect on disability of possible ligament injury after distal radius fracture.

An advantage of our study is that we recorded baseline DASH score as a measure of disability before fracture. This reduces the risk of bias in cases of comorbidity. The DASH score at baseline relied on patient recall, as the questionnaires were sent to the patients within one week of fracture; a procedure previously reported [30]. Although patients could have misunderstood the intention of responding to the baseline DASH questionnaire and answered on the basis of current status (i.e. *with *fracture), we believe that this cannot be a substantial problem since baseline DASH in our data was very low. Since the DASH questionnaire is self-administered uncertainty regarding accurate completion of the questionnaire is a potential problem. The DASH has however been widely used as a mailed questionnaire. Questionnaires with more than three unanswered questions were excluded.

## Conclusion

We concluded that after a displaced extraarticular or intraarticular distal radius fracture, treated with closed reduction and cast or with external or percutaneous pin fixation, malunion in terms of a dorsal tilt exceeding 10 degrees and/or a positive ulnar variance was associated with higher arm-related disability at one year after injury, regardless of patient age or gender.

## Competing interests

The authors declare they have no competing interests.

## Authors' contributions

EB contributed to study conception, analysis and interpretation of data and drafting of the manuscript; MH and MP participated in acquisition of data, PW participated in analysis and interpretation of data, LD participated in critical revision of the manuscript; IA participated in study conception and design and critical revision of the manuscript. All authors read and approved the final manuscript.

## Pre-publication history

The pre-publication history for this paper can be accessed here:

http://www.biomedcentral.com/1471-2474/12/9/prepub
